# Perioperative use of intra-articular steroids during the COVID-19 pandemic

**DOI:** 10.1007/s00590-021-03105-x

**Published:** 2021-09-01

**Authors:** Eric Jou, Andrew Kailin Zhou, Jamie Sin Ying Ho, Azeem Thahir

**Affiliations:** 1 School of Clinical Medicine, University of Cambridge, Cambridge, UK; 2 MRC Laboratory of Molecular Biology, Cambridge Biomedical Campus, Cambridge, UK; 3 North Middlesex University NHS Trust, London, UK; 4 Department of Trauma and Orthopaedic Surgery, Addenbrooke’s Hospital, Cambridge University Hospitals NHS Foundation Trust, Cambridge, UK

**Keywords:** Injections, Intra-articular, Perioperative period, Steroids, SARS-CoV-2, COVID-19, Pandemics

## Abstract

**Purpose:**

There are growing concerns with the widely used glucocorticoids during the Coronavirus disease-19 (COVID-19) pandemic due to the associated immunosuppressive effects, which may increase the risk of COVID-19 infection and worsen COVID-19 patient outcome. Heavily affecting orthopaedics, the pandemic led to delay and cancellation of almost all surgical cases, and procedures including perioperative intra-articular corticosteroid injections (ICIs) saw similar decreases. However, the benefits of ICI treatments during the pandemic may outweigh these potential risks, and their continued use may be warranted.

**Methods:**

A literature search was conducted, and all relevant articles including original articles and reviews were identified and considered in full for inclusion, and analysed with expert opinion. Epidemiological statistics and medical guidelines were consulted from relevant authorities.

**Results:**

ICIs allow a targeted approach on the affected joint and are effective in reducing pain while improving functional outcome and patient quality-of-life. ICIs delay the requirement for surgery, accommodating for the increased healthcare burden during the pandemic, while reducing postoperative hospital stay, bringing significant financial benefits. However, ICIs can exert systemic effects and suppress the immune system. ICIs may increase the risk of COVID-19 infection and reduce the efficacy of COVID-19 vaccinations, leading to important public health implications.

**Conclusion:**

Perioperative ICI treatments may bring significant, multifaceted benefits during the pandemic. However, ICIs increase the risk of infection, and perioperative COVID-19 is associated with mortality. The use of ICIs during the COVID-19 pandemic should therefore be considered carefully on an individual patient basis, weighing the associated risks and benefits.

**Supplementary Information:**

The online version contains supplementary material available at 10.1007/s00590-021-03105-x.

## Introduction

The use of intra-articular corticosteroid injections (ICIs) started in the early 1950s [1] and is now routinely used for management of joint pain and therapeutic treatment across a broad range of conditions [2–4]. ICI treatments allow symptomatic relief and show therapeutic efficacy when administered to almost all major areas of the body, including the joints of the upper limb (shoulder, elbow, wrist and hand), lower limb (knee, ankle and foot), facet joints of the spine, the sacroiliac joint, the coccyx, and the hip, leading to its widespread use with broad implications [4]. In particular, perioperative ICIs are commonly used for pain relief, allowing better recovery of function postoperatively and improved patient quality-of-life [5, 6]. Perioperative is defined as the medical care from the time of considering surgery through to the postoperative period where the patient strives to obtain a full recovery (preoperative, intraoperative and postoperative) [7]. Nevertheless, due to the potential danger of infections associated with steroid-mediated immunosuppression [8–12], the contemporary COVID-19 pandemic has instigated significant additional risks that need to be addressed when considering ICI use.

The Coronavirus disease-19 (COVID-19) pandemic is caused by the severe acute respiratory syndrome coronavirus 2 (SARS-CoV-2), a novel coronavirus transmitted via respiratory droplets, and was first identified in late 2019 in Wuhan, China [13]. As estimated by the WHO, there are more than 110 million confirmed cases globally, with 2.5 million deaths worldwide as of February 2021 [14]. Disease severity in COVID-19 patients ranges from asymptomatic to life-threatening, with severe disease and mortality being more common in the elderly and in patients with underlying health conditions [15]. Furthermore, a significant proportion of COVID-19 patients may continue to experience long-term detrimental effects systemically, affecting the lungs, heart, brain, and the immune system [16], several weeks or months after the initial infection. The pandemic imposed an unprecedented burden on healthcare worldwide, forcing healthcare provision and practice to adapt globally [17–19]. In particular, the use of immunosuppressive agents, including the widely used corticosteroids, may heighten the risk of viral infections and increase COVID-19 severity [15, 20–23]. Perioperative use of ICIs must be carefully evaluated, and the impact on patient outcome needs to be established.

Surgical procedures have been particularly affected by the COVID-19 pandemic [24, 25]. Many countries made the difficult decision to postpone or cancel most elective surgeries, to better direct medical personnel and resources to combat the pandemic [26, 27]. In particular, orthopaedic procedures have been heavily impacted. Over a 12-week period of peak healthcare disruption, a projected 28.4 million surgical operations would be cancelled globally, of which 6.3 million are non-emergency orthopaedic surgeries [24]. Orthopaedics is predicted to be the most heavily affected surgical specialty worldwide, with the highest cancellation rate (at 82%) out of all surgical specialties. Hospital beds are prioritised for COVID-19 patients, and surgical trainees are redeployed to reinforce medical specialties in treating COVID-19 patients [28], including orthopaedic trainees across the UK [29, 30]. As of February 2021, 10 million patients are awaiting surgery in the UK, a dramatic increase from 4 million pre-pandemic [26]. Recognising the growing concerns of large-scale cancellation of non-emergency procedures [31, 32], elective surgeries had resumed since the latter half of 2020, albeit at much reduced capacities [26]. Healthcare services, and how perioperative care is delivered, had to adapt accordingly to provide the best care for patients. Perioperative ICIs may play an important role in improving symptom control and patient quality-of-life during the delay to elective surgeries, for example for elective joint replacements commonly performed for arthritis. Importantly, reassessing the safety and efficacy of these common perioperative management strategies employed prior to the pandemic, including ICIs, to adapt to the current pandemic is paramount.

The aim of this article was to review the benefits and risks associated with perioperative ICIs during the COVID-19 pandemic in light of recent evidence, with specific focus on the impact by the pandemic, along with medical, financial and public health implications. The potential interactions between ICIs and the immune response against SARS-CoV-2 is also discussed and is of high relevance with important impact on the ongoing COVID-19 vaccination programme.

### Methods

A literature search was first conducted on PubMed in January 2021, and again in February 2021, and all the relevant articles in the English language, including original articles and reviews, were identified. All non-peer-reviewed articles were excluded. Key search terms used were “steroid”, “intra-articular injection”, “COVID-19”, “SARS-CoV2”, “pandemic”, “virus”, “immune”, and “vaccine”. No articles were excluded based on publication date. A serial screening method was adopted due to the large number of candidate articles. Initially, article titles were screened for relevance to the factors discussed in this narrative review, followed by a second round of screening through the abstracts. Relevant articles were then considered in full, and the references cited in these articles were additionally screened, before inclusion. The World Health Organization (WHO) website was consulted for up-to-date global statistics regarding the COVID-19 pandemic and recommended guidelines on steroid use during the pandemic. The UK government website, and websites of relevant healthcare bodies including the National Health Service (NHS), the National Institute for Health and Care Excellence (NICE), were consulted for COVID-19 pandemic-related healthcare statistics, healthcare guidelines, and details on the UK COVID-19 vaccination programme. The PRISMA flow diagram for the literature search is presented in the supplementary material.

### ICI treatments during the COVID-19 pandemic

ICI treatments involve the administration of glucocorticoids, a class of corticosteroids that binds to the glucocorticoid receptor. Glucocorticoids were first used therapeutically in humans in 1948 to treat rheumatoid arthritis (RA) [33], and ICIs are now commonly used in clinical practice [34]. Clinically administered glucocorticoids are similar in structure and function to endogenous cortisol, exerting potent anti-inflammatory effects in a dose-dependent manner [35]. Due to their widespread action on various cell types, clinical use of glucocorticoids has been limited by the broad range of potential adverse effects. In particular, the pioneering study by Fauci et al. in the 1970s identified that glucocorticoid administration leads to lymphopenia and immunosuppression [36], and steroid use is associated with increased risk of infection both in the community and in hospital settings [8–12]. Importantly, the increased risk of infection is not limited to systemic steroid use, with growing evidence demonstrating that local administration of steroids also heightens infection risk [37, 38]. Accordingly, in patients with rotator cuff tendinosis, a single corticosteroid injection up to a month prior to surgery increased the risk of postoperative infections twofold [37]. The use of steroids in the contemporary COVID-19 pandemic thus needs to be exercised with caution.

Regulating bodies including the National Health Service (NHS), the British Orthopaedic Association, and the Faculty of Pain Medicine, have raised concerns on the immunosuppressive effects of corticosteroids, issuing specific guidance accordingly to limit the use of steroid injections during the COVID-19 pandemic [39–41]. Clinicians are advised to maintain awareness of the risks associated with corticosteroid injections during the COVID-19 pandemic, and to assess the risk to benefit ratio for each patient on an individual basis. Specifically, ICIs should only be used in inflammatory conditions where there is active synovitis and at the lowest clinical effective dose. For musculoskeletal pain management, ICIs may only be used if other methods, including simple analgesia, splinting, active modification, and exercise prove ineffective, in addition to a high degree of pain and disability projected to have a significant impact on patient health and wellbeing [40]. Strictly, injected steroids should not be administered in the presence of an infection.

The guidance is welcomed, as studies suggest steroids are often misused clinically without definitive evidence on safety and efficacy [42, 43]. Often underappreciated, local administration of steroids can exert systemic effects [44], where a single dose of 40 mg of methylprednisolone is sufficient for complete hypothalamic–pituitary–adrenal (HPA) suppression. This is well within the range of 4-80 mg methylprednisolone routinely used clinically through ICIs [45]. Others have similarly found that a single dose of ICI can suppress the HPA axis systemically [46, 47]. Strikingly, increased duration of systemic suppression is observed after ICI treatment compared to oral steroids, due to the slower rate of absorption through joint tissue [48]. A 46% mean decrease in both C-reactive protein (CRP) and erythrocyte sedimentation rate (ESR) was observed in RA patients after knee ICI treatment, with the effect lasting up to 6 months [49]. Independent studies had found a similar decrease in CRP or ESR levels in RA patients post ICI treatment [50, 51], suggesting systemic anti-inflammatory effects of ICIs. In line with this, ICIs decrease pro-inflammatory cytokines and inhibit monocyte function systemically [52, 53] and increase the risk of infection after knee, hip and shoulder arthroscopy [38]. Given that ICIs may lead to systemic immunosuppression and increase risk of infection, it should be used with caution clinically, more so amid the COVID-19 pandemic. Further studies are required to better understand the interactions between ICI-mediated immunosuppression and COVID-19 infections.

### ICIs and the immune response against SARS-CoV-2

**Fig. 1:**
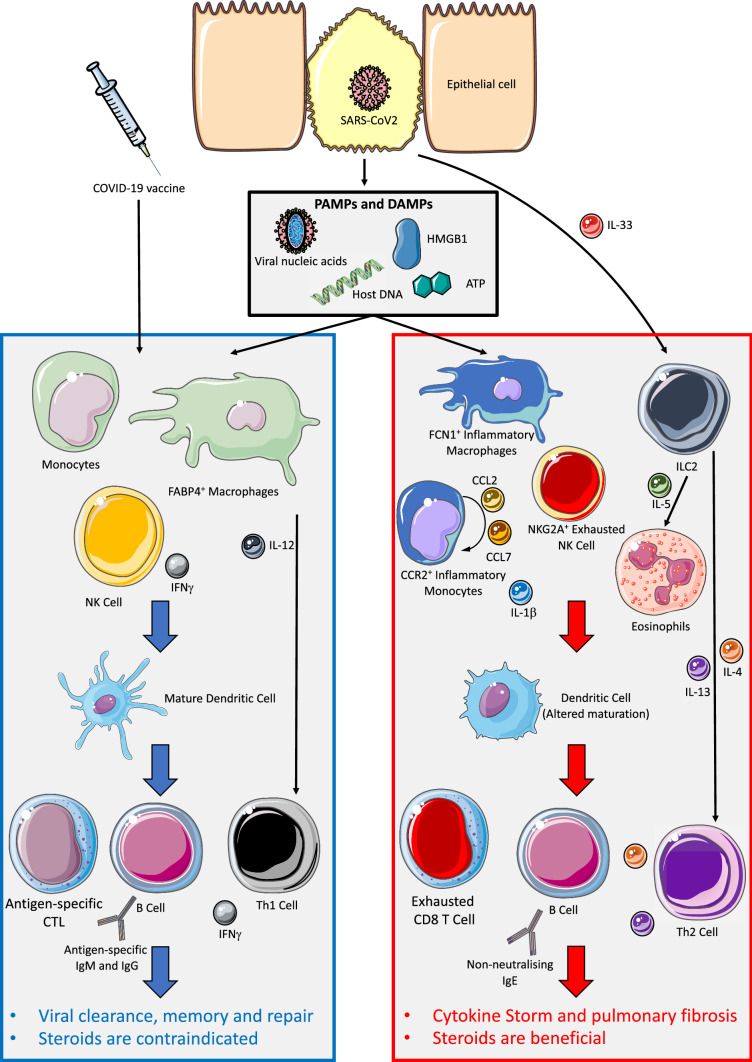
The immune response dichotomy against SARS-CoV2 infection in airway epithelial cells leads to cell death and the release of damage-associated molecular patterns (DAMPs), including adenosine triphosphate (ATP), DNA, and High Mobility Group Box 1 (HMGB1), the latter elevated in COVID-19 patients and correlates with inferior outcome. DAMPs and SARS-CoV2-derived pathogen-associated molecular patterns (PAMPs) including viral nucleic acids are detected by tissue resident innate immune cells such as macrophages, leading to cytokine production and recruitment of monocytes and NK cells which further amplifies the response. In mild and moderate COVID-19 patients (left blue panel), FABP4^+^ alveolar macrophages and anti-viral NK cells produce IL-12 and interferon-γIFNγ, respectively, and together with mature dendritic cells, promote CD4 T cell T helper type 1 (Th1) polarisation and activation of antigen-specific CD8 cytotoxic T lymphocytes (CTLs), leading to elimination of viral-infected cells. B cells produce antigen-specific IgM and IgG, which are effective in neutralising the virus, and together, this is characteristic of an effective anti-viral type 1 immune response, allowing viral clearance and development of immunological memory. A similar response is observed after COVID-19 vaccination in non-COVID-19 patients. In severe COVID-19 patients (right red panel), an inappropriately heightened immune response leads to a significant increase in CCR2^+^ inflammatory monocytes and FCN1^+^ inflammatory macrophages, replacing the FABP4^+^ alveolar macrophages seen in non-severe patients. A positive-feedback loop through increased production of chemokines CCL2 and CCL7 further recruits inflammatory monocytes and promote the production of pro-inflammatory cytokine IL-1γ. In these patients, NK cells show an exhausted phenotype, characterised by NKG2A expression, and have impaired anti-viral function. Additionally, widespread epithelial damage releases the alarmin IL-33, leading to the characteristic increase in group 2 innate lymphoid cells (ILC2s) seen in severe COVID-19 patients. ILC2s are potent producers of IL-4, IL-5 and IL-13. IL-5 recruits and activates eosinophils, and increased eosinophils contribute to the inflammatory pathology and correlates with adverse outcomes in COVID-19 patients. Altered dendritic cell maturation, together with the IL-4 and IL-13 produced by ILC2s, lead to altered CD4 T cell activation and subsequent polarisation towards a T helper type 2 (Th2) phenotype with impaired anti-viral functions. Th2 cells further produces IL-4 and IL-13, which promotes B cell antibody class switch to IgE, and the non-neutralising antibodies may facilitate SARS-CoV2 infection via antibody-dependent enhancement. CD8 T cells show an exhausted phenotype with defective anti-viral functions. Together, the heightened inflammatory environment and inadequate viral clearance can result in irreversible pulmonary fibrosis and perpetuate a cytokine storm, leading to multi-organ damage. In severe COVID-19 patients, steroids reduce mortality by dampening the inappropriately hyperactivated immune response and cytokine storm, while its use is contraindicated in mild and moderate COVID-19 patients where suppression of the protective anti-viral immune response may increase disease severity. In non-COVID patients, steroids may increase the risk of infection and decrease the efficacy of COVID-19 vaccinations [54, 57–59, 64, 69, 70, 99, 102–105]. This figure was created using templates by Servier Medical Art, licensed under a Creative Commons Attribution 3.0 Unported License (https://smart.servier.com/)

The immune response against SARS-CoV-2 is complex (Fig. 1), and despite the rapid advances in recent months, much remains to be clarified. Accumulating evidence suggests that both the innate and the adaptive immune systems play crucial roles in the defence against SARS-CoV-2 [54], and better understanding of these processes is vital to improve patient care and outcome amid the pandemic. Specifically, it is crucial to elucidate the impact of corticosteroids on the immune response against SARS-CoV-2.

The immune response is effective in clearing SARS-CoV-2 infections in most patients, with an overall estimated death rate of 0.66% [55]. However, mortality rises sharply to 40% in patients with severe COVID-19 [56], where abnormal immune hyperactivation inflicts indirect host damage through widespread lung inflammation and multi-organ damage [54]. The reason behind this divergence in patient immune response against SARS-CoV-2 is unclear, but likely relates to the immune dysregulation observed in severe COVID-19 patients. Increase in inflammatory monocytes correlates with severity of disease [57, 58], and recruitment is through chemokines CCL2 and CCL7, both of which elevated in fatal COVID-19 cases [59]. Monocytes directly contribute to the cytokine storm profile observed in severe COVID-19 patients, indicated by a systemic increase in interleukin-6 (IL-6), interleukin-8 (IL-8), interferon-γ inducible protein 10 (IP-10), monocyte chemoattractant protein-1 (MCP1), macrophage inflammatory protein-1 alpha (MIP1γ, and TNF [54, 60]. In line with this, anti-inflammatory therapies through immunosuppressive glucocorticoids decrease mortality in severe COVID-19 patients and should be used [61, 62]. Any non-emergency surgery for patients that develop severe COVID-19 must be postponed and only proceed when medically safe to do so.

While steroid use is advocated in severe COVID-19 cases [63], its use in non-COVID-19 patients or in mild and moderate COVID-19 cases should be exercised with caution, due to the immunosuppressive effects as indicated by the WHO [64]. In mild and moderate COVID-19 patients, steroid use correlates with prolonged duration of fever, delayed virus clearance time, severe disease and longer hospitalisation [65–68]. Mechanistically, antigen-specific type 1 immunity driven by T helper type 1 (Th1) and CD8 T cells are paramount in mounting a strong multimodal immune response against SARS-CoV-2 (Fig. 1), characterised by robust interferon-γ (IFNγ) production and a strong B cell-mediated antibody response [57, 69]. Conversely, patients that develop severe COVID-19 have decreased T cells, and T cells show an exhausted phenotype with impaired functions [70]. Reduced lymphocyte count is a negative prognostic indicator in COVID-19 patients [71]. Given that a single dose of ICI reduces systemic blood lymphocyte count, leading to lymphopenia four hours after injection [52], ICIs may impair the host protective response against SARS-CoV-2. The systemic and immunosuppressive effects of steroid injections may last several weeks [72] and could consequentially expose patients to COVID-19 infection and developing severe disease. Accordingly, ICIs increases the risk of influenza even among vaccinated individuals [23]. In this study, the average dose patient received through a single ICI was 65.9 mg, with greater than half of the patients receiving > 80 mg of methylprednisolone or triamcinolone acetonide or equivalent, which is well within the range to cause systemic effects [44]. Critically, the risk of patients acquiring COVID-19 infection perioperatively should be minimised, as perioperative SARS-CoV-2 infection increases mortality in both elective and emergency surgical patients [73]. The National Institute for Health and Care Excellence (NICE) published guidelines for elective patients to follow comprehensive social-distancing measures for 14 days, and to test for SARS-CoV-2 within 3 days prior to hospital admission [74]. The threshold for surgery should be heightened during the COVID-19 pandemic, and use of perioperative ICI must be carefully evaluated. The increased risk associated with COVID-19 infection must be balanced with the consequences of delaying elective surgeries, and the use of ICIs should be discussed in conjunction with patients on an individual basis.

### Impact on patient quality-of-life during the COVID-19 pandemic

In light of the adverse effects of systemic glucocorticoid treatments, ICI is popular as it allows a localised approach with targeted effect on the affected joint [75]. Perioperative ICIs are common for a wide range of orthopaedic surgeries, both pre- and postoperatively.

### Preoperative use of ICI during COVID-19: delaying elective surgery

In osteoarthritis (OA) patients requiring surgery, preoperative ICIs are associated with a delay to TKA from first presentation to clinic by 8 months, and a similar 5-month delay for patients that received total hip arthroplasty [76]. This is attributed to preoperative ICIs providing comfort, symptom alleviation and reducing functional disability. Overall, the benefits of the delay in time to surgery from initial clinical presentation are twofold. Firstly, it allows patients time to prepare for surgery mentally and to make necessary arrangements for social support. Secondly, and specifically related to the COVID-19 pandemic where elective surgeries are postponed, preoperative ICIs are effective in improving patient quality-of-life during the delay to surgery. Reassuringly, studies of patients given ICI during the COVID-19 pandemic in the UK did not show any increase in COVID-19 infection or adverse effects [77, 78]; therefore, ICI may be a safe and viable option. Of note, while ICI clinics may also work at reduced capacities during the pandemic, reinstating ICI clinics could be a practical and more feasible approach to combat the significant delays in elective surgeries.

### Postoperative use of ICI during COVID-19: post-operative pain management

Postoperative ICIs reduce pain and swelling and improve range of motion after TKA [6]. Improving patient quality-of-life is paramount, as 50% of patients suffering from chronic pain have associated psychological distress [79]. OA is one of the top ten most debilitating diseases worldwide [80], causing significant pain with 60% of patients awaiting knee replacement surgery showing signs of clinical depression [26]. Clinical depression may further rise to 90% in patients suffering from orthopaedic-related axial pain. Patients diagnosed with mental disorders have increased risk of acquiring COVID-19 [81] and are more likely to develop severe disease [82]. Furthermore, severe postoperative pain is associated with suppressed immune function, and may predispose to infection [83]. Use of ICIs may improve patient health both physically and mentally and thus achieve better quality-of-life amid the pandemic and should be considered after discussing and assessing each patient and their specific circumstances on an individual basis.

There is an inherent fear for a potential rise in self harm or suicide rate during the COVID-19 pandemic. While current existing reports had found no increase in suicide rates in relation to the pandemic [84], it is essential to remain vigilant, as a potential increasing trend in certain vulnerable groups may be masked by an overall unchanging suicide rate. Strikingly, patients with arthritis have significantly increased risk for suicide with 46% higher odds than those without arthritis [85]. Among the arthritis patients, those currently experiencing chronic pain had increased risk of suicide compared to those without pain (odds ratio of 1.5). The delay in elective surgeries due to the pandemic is unprecedented and can lead to prolonged suffering of pain in arthritis patients awaiting joint replacement surgeries. The American Academy of Orthopaedic Surgeons had suggested that preoperative ICIs should be considered for pain management in surgical arthritis patients during the delay to surgery [86]. The use of ICIs can improve pain management and may be effective in reducing suicide rates in arthritis patients. Extended suffering during the delay to surgery for elective patients should not be forgotten, and increasing the capacity of ICI clinics during the pandemic should be considered as a feasible temporary solution. Better symptomatic control and pain management is more important than ever amid the pandemic, and ICIs are an effective treatment modality that can be employed to achieve this.

### Financial implications of perioperative ICIs amid the COVID-19 pandemic

The COVID-19 pandemic had imposed significant financial burden on healthcare systems worldwide, with an estimated £40 billion extra healthcare service cost per year on the NHS [87]. The cost-effectiveness of treatment modalities needs to be considered. A recent UK-based study concluded that addition of ICI and lidocaine to standard hip OA treatment significantly improved function and incurred less cost (£161.59 per patient) over a 6-month period [88], attributed to the higher quality-adjusted life-years. In a randomised control trial (RCT), sonographic guidance for knee ICIs reduced procedural pain, doubled the responder rate, increased the therapeutic duration by 36% while reducing cost by 58% in responders per year [89]. Use of sonographic guidance over traditional anatomy landmark methods during ICIs directly improves clinical outcome and cost-effectiveness and should be used whenever appropriate [90]. Another UK study found steroid injections to be cost-effective compared to physiotherapy, with an average saving of £43.32 per patient while outcomes were similar [91]. Alternatively, intra-articular injection of ketorolac produced similar degree of pain relief to ICI while saving cost in a small study and may be considered [92]. Strikingly, perioperative corticosteroid injection prior to TKA shortens mean hospital stay by 25% in a RCT [93]. This is desirable as reducing hospitalisation time decreases the risk of hospital-acquired COVID-19, accommodates for the increased medical demand amid the pandemic [94], and has important financial implications on cost-savings from reduced hospital stay [95, 96].

### Public health implications of ICI treatments on the COVID-19 vaccination programme

Global roll-out of COVID-19 vaccines plays a critical role in combating the pandemic. The comprehensive, tiered UK vaccination programme prioritises the most clinically vulnerable, to protect those that are at highest risk of severe COVID-19 outcomes [97]. Factors associated with worse COVID-19 prognosis, including reduced physical activity, increased age and body mass index, are associated with orthopaedic conditions such as OA where ICIs are routinely used [98]. Of concern, ICIs may reduce the efficacy of COVID-19 vaccinations at clinically used doses [99], due to the potential immunosuppressive effects on the protective type 1 immune response (Fig. 1), observed both in natural COVID-19 infections [57] and after COVID-19 vaccination [69]. While more studies are required to assess the specific effects of ICIs on COVID-19 vaccinations, previous studies identified ICIs to reduce the efficacy of influenza vaccination [23]. Suboptimal response to COVID-19 vaccination will have significant negative public health implications and should be avoided at all costs. Current guidance in the UK suggests that for elective patients, COVID-19 vaccinations should take priority over any form of steroid injections including ICIs, which is advised to be postponed by 2 weeks to allow optimal response to the COVID-19 vaccine [99]. Finally, it has been suggested that evolution of SARS-CoV2 may be accelerated in immunosuppressed individuals, due to the suboptimal immune response allowing viral persistence [100]. Furthermore, existing COVID-19 therapies, such as remdesivir, may be less effective in eradicating SARS-CoV2 in immunosuppressed individuals leading to persistent infections [101], which may potentially facilitate the appearance of vaccine escape strains.

## Conclusion

**Fig. 2:**
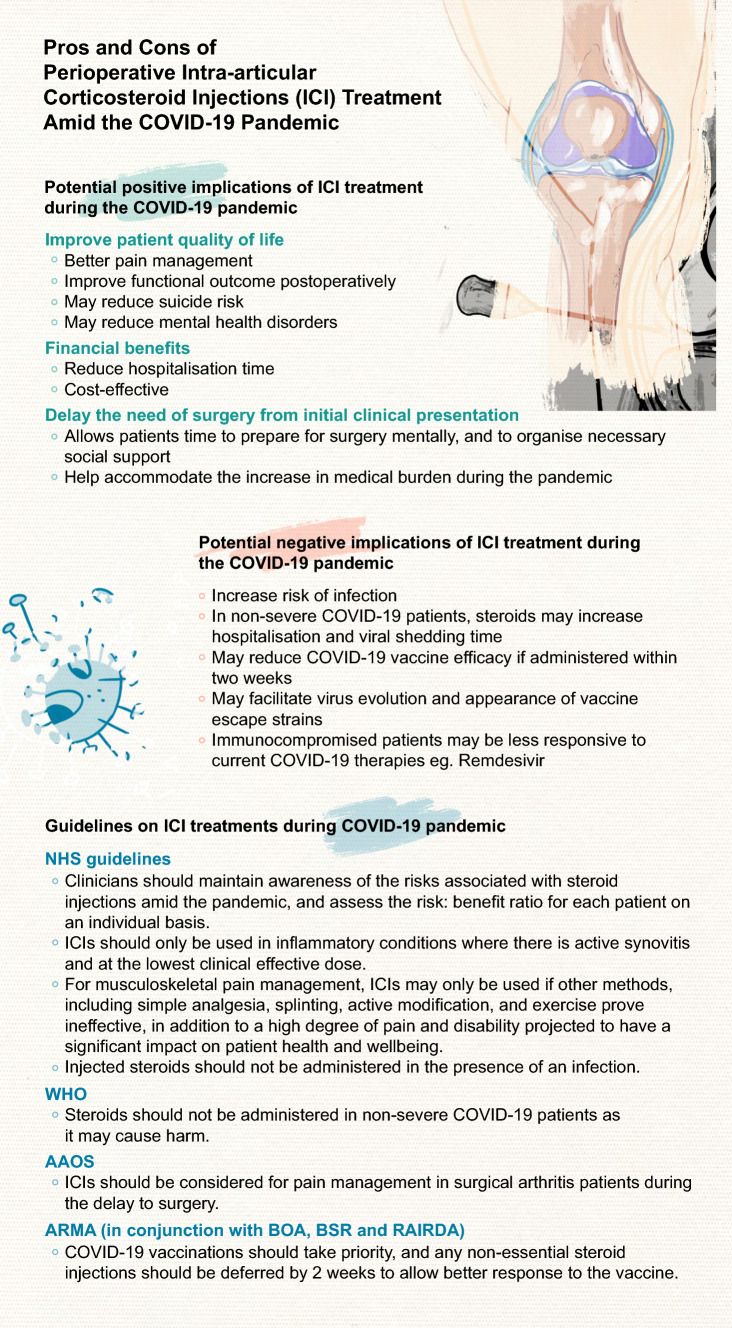
Implications of intra-articular corticosteroid injections amid the Coronavirus disease-19 (COVID-19) pandemic. The positive and negative implications of ICI treatment during the COVID-19 pandemic, along with recent guidelines by relevant authorities, are summarised in the infographic

In accordance to recent guidance from the NHS, the threshold for perioperative ICIs should be heightened amid the COVID-19 pandemic. Although administered locally, ICIs exert systemic anti-inflammatory and immunosuppressive effects at clinically relevant doses and increase the risk of perioperative infections. Importantly, perioperative SARS-CoV-2 infection should be avoided due to the associated increase in mortality. ICIs may reduce the efficacy of COVID-19 vaccinations, which can have significant public health impacts. Equally, while capacities for elective ICI procedures during the pandemic may be reduced, reinstating perioperative ICIs to improve pain management and patient quality-of-life is suggested by the American Academy of Orthopaedic Surgeons. ICI treatments may delay the need for surgery, while enhancing functional outcome postoperatively, and may be cost-effective with important financial implications. Arthritis patients have increased risk of suicide, and suicide rate is further increased in those suffering from chronic pain. The delay in elective surgeries instigated by the pandemic may prolong patient suffering in those awaiting joint replacement surgeries. ICIs may allow better symptomatic control and pain management during the delay. Of note, poorly managed postoperative pain is associated with immunosuppression and worse functional outcome. Clinical decisions on the use of ICIs should thus be made on an individual basis, and discussed with patients to achieve the best overall outcome (Fig. 2). It is paramount that flexibilities exist in the guidelines for ICI treatments to best accommodate for individual patient circumstances. Future research will provide further evidence to better evaluate the safety of ICI treatments during the COVID-19 pandemic.

## Supplementary Information

Below is the link to the electronic supplementary material.

Supplementary file1 (DOCX 34 kb)

## Declarations

### Data Availability

Data available upon request.
