# Supplementary material for: Perioperative use of intra-articular steroids during the COVID-19 pandemic
Source: Eur J Orthop Surg Traumatol. 2021 Sep 1;32(7):1225–35. doi: 10.1007/s00590-021-03105-x (PMC8408365; doi:10.1007/s00590-021-03105-x)
Supplement: Supplementary file 1 — Supplementary file1 (DOCX 34 kb) [file 590_2021_3105_MOESM1_ESM.docx]

**Identification of studies via databases and registers**

Records identified from*:

Medline n= 1749

*Note search strategy limited to humans in database

**Identification**

Records excluded

(n = 1308)

Records screened

(n = 1749)

**Screening**

Reports assessed for eligibility

(n = 441)

Reports excluded:

Not relevant (n = 347)

**Included**

Studies included in review

(n = 94)
